# Australian children’s consumption of caffeinated, formulated beverages: a cross-sectional analysis

**DOI:** 10.1186/s12889-015-1443-9

**Published:** 2015-01-31

**Authors:** Kelsey Beckford, Carley A Grimes, Lynn J Riddell

**Affiliations:** Centre for Physical Activity and Nutrition Research, School of Exercise and Nutrition Sciences, Deakin University, 221 Burwood Highway, Burwood, Melbourne, Victoria 3125 Australia

**Keywords:** Caffeine, Dietary, Beverages, Dietary, Soft drink, Child, Adolescent, Australia

## Abstract

**Background:**

Caffeine is a common additive in formulated beverages, including sugar-sweetened beverages. Currently there are no data on the consumption of caffeinated formulated beverages in Australian children and adolescents. This study aimed to determine total intake and consumption patterns of CFBs in a nationally representative sample of Australian children aged 2–16 years and to determine contribution of CFBs to total caffeine intake. Consumption by day type, mealtime and location was also examined.

**Methods:**

Dietary data from one 24-hour recall collected in the 2007 Australian National Children’s Nutrition and Physical Activity Survey were analysed. CFBs were defined as beverages to which caffeine has been added as an additive, including cola-type beverages and energy drinks. Socioeconomic status was based on the highest level of education attained by the participant’s primary caregiver. Time of day of consumption was classified based on traditional mealtimes and type of day of consumption as either a school or non-school day. Location of consumption was defined by the participant during the survey.

**Results:**

On the day of the survey 15% (n = 642) of participants consumed CFBs. Older children and those of low socioeconomic background were more likely to consume CFBs (both P < 0.001). Amongst the 642 consumers mean (95% CI) intakes were 151 (115–187)g/day, 287 (252–321)g/day, 442 (400–484)g/day, and 555 (507–602)g/day for 2–3, 4–8, 9–13 and 14–16 year olds respectively. Consumers of CFBs had higher intakes of caffeine (mean (95% CI) 61 (55–67)mg vs. 11 (10–12)mg) and energy (mean (95% CI) 9,612 (9,247-9978)kJ vs. 8,186 (8,040-8,335)kJ) than non-consumers (both P < 0.001). CFBs contributed 69% of total daily caffeine intake. CFB intake was higher on non-school days compared with school days (P < 0.005) and consumption occurred predominantly at the place of residence (56%), within the “dinner” time bracket (17:00–20:30, 44%).

**Conclusions:**

The consumption of CFBs by all age groups within Australian children is of concern. Modifications to the permissibility of caffeine as a food additive may be an appropriate strategy to reduce the intake of caffeine in this age group. Additional areas for intervention include targeting parental influences over mealtime beverage choices.

## Background

Caffeine, 1,3,7-trimethylxanthine, is a naturally occurring alkaloid found in plant materials such as coffee beans, tea leaves, cola nuts and cacao beans [1]. Caffeine is a common additive in formulated beverages, including sugar-sweetened beverages (SSBs), artificially-sweetened beverages (ASBs) and energy drinks [2].Variations in the regulation of caffeine’s addition to beverages exist internationally, with countries differing in both the allowable concentration of caffeine in beverages (ranging 150 mg/L-320 mg/L) and the types of beverages to which it can be added [2-5]. Addition of caffeine to commercial beverages in Australia is strictly regulated by Food Standards Australia New Zealand (FSANZ), with no more than 145 mg/kg permitted in cola-type formulated beverages [6], and 320 mg/L in energy and sports drinks [7]. In response to the increase in caffeine consumption being observed internationally [8], food regulatory authorities in Australia and New Zealand [9], Canada [9] and the United States [10] are currently reviewing their country specific regulations concerning caffeine as an additive to food products.

Both the naturally occurring and artificial forms of caffeine exert similar physiological effects on humans, as there is no difference in the chemical structure between the two forms [1]. In modest doses of up to 400 mg, caffeine consumption has been associated with positive post-ingestive effects on mood, alertness and performance in adults [11]. However consumption at doses above these levels may result in increased anxiety levels as well as decreased physical and cognitive performance [12]. Currently there are no recognised health-based guidance values on recommended daily intakes for caffeine in Australia and New Zealand, however a recent review by the FSANZ caffeine expert working group found that doses of caffeine at approximately 3 mg per kilogram of bodyweight led to increased anxiety levels in children [2]. This is similar to recommendations made by Health Canada that children under twelve years of age should not consume more than 2.5 mg per kilogram of bodyweight [9]. Using the current Australian National Health and Medical Research Council’s reference ranges for bodyweight [13], this would equate to approximately 39–171 mg of caffeine for 2–16 year olds.

Studies from the UK, US and Canada in children and adolescents have shown that formulated beverages are the most commonly consumed form of caffeine amongst children and adolescents [14-16]. This is concerning as these beverages are often energy-rich, and nutrient poor [17], and have been linked with adverse health outcomes in children and adolescents, including dental caries [18], bone health [19], and in the case of SSBs, overweight and obesity [20-23]. Manufacturers claim that caffeine is added formulated beverages, including ASBs and SSBs, as a flavouring agent [24]. This has been questioned by researchers, who have determined that most consumers are unable to detect caffeine in beverages on the basis of taste [17,25]. Hence it has been hypothesised that caffeine is added to beverages in order to promote repeat consumption, due to the mildly addictive nature of the caffeine compound [26]. Repeat consumption of energy-rich, nutrient poor SSBs, promoted by caffeine, could potentially lead to increased quantities being consumed, and, in turn, an increased risk of adverse health outcomes [26,27].

At present there are no data available regarding intake of caffeinated, formulated beverages (CFBs) in Australian children and adolescents. Given the potential adverse health outcomes associated with high intakes of these beverages, monitoring of CFB intake in children is important. The aims of this study were: i) to determine intake of CFBs, where caffeine has been added as an ingredient, and subtypes of these beverages (i.e. ASBs, SSBs, and energy drinks) in a nationally representative sample of Australian children aged 2–16 years; ii) to determine the contribution of these beverages to total caffeine intake and; iii) to examine where (location) and when (meal times and school day vs. weekend day) CFBs are consumed.

## Methods

### Study design

This study used data collected from the 2007 Australian Children’s Nutrition and Physical Activity Survey (CNPAS). CNPAS was a cross-sectional study on children’s dietary and physical activity behaviours. Full details of the CNPAS sampling and survey methodologies have been previously reported [28], as has our own analysis of these data [29]. In brief, a nationally representative sample of 4,487 children aged 2–16 years were recruited for the survey, using a multistage quota sampling framework [28]. Data were collected between February and August 2007. Ethics was approved for this survey by the National Health and Medical Research Council Registered Ethics Committees of Commonwealth Scientific and Industrial Research Organisation and the University of South Australia. For all participants consent was obtained from the primary caregiver. In addition, in those aged 14–16 years assent was obtained [28].

### Anthropometry

Height and weight were collected using standardized protocols [28]. Body Mass Index (BMI) was calculated as weight (kg) divided by height (m^2^). Weight status was determined using the International Obesity Task Force cut-off points for BMI for children [30,31].

### 24-hour dietary recall

Dietary data were collected using a computer assisted 3-pass 24-hour dietary recall, during which time all data pertaining to the time and location of consumption of each food and beverage item were collected [28]. Portion sizes were estimated by using a validated food model booklet and standard household measures. The 24-hour dietary recall was conducted with the primary caregiver of participants aged <9 years and with the study child in participants aged 9 years and over [28]. The three-pass dietary recall method is a standardised procedure used in Australian and New Zealand national nutrition surveys, including a national survey of New Zealand schoolchildren 5–14 years of age [32]. Further details of the dietary collection methodology can be found in the CNPAS user guide [28]. Energy and caffeine intakes were determined using the AUSNUT2007 nutrient composition database, which was specifically developed for the CNPAS [33].

### Definition of day type, meal times and location of consumption

The type of day on which each participant recalled their dietary intake for was recorded as a weekday, weekend, public holiday or school holiday. In the current analysis non-school days were defined as a weekend, public holiday or school holiday day and school days were defined as a weekday. In the absence of participant defined meal times, classification was based on the time of reported consumption. Meal times were defined as follows: breakfast: 05:00 – 08:59, morning tea 09:00 – 11:29, lunch: 11:30 – 13:59, afternoon tea: 14:00 – 16:59, dinner: 17:00 – 20:29 and supper: 20:30 onwards. Location of consumption was defined by the participant during the 24 hour recall as either: i) place of residence: incorporating “home” and “other residence”, for example a family member or friend’s home, ii) place of purchase, iii) institution, iv) during a leisure activity, v) during transport and vi) other/no data available.

### Beverage classification

The CNPAS food group coding system [28] was used to classify beverage items consumed during the survey. All food items consumed on the day of the survey were assigned an eight-digit food code, which linked each food item to nutrient information within the AUSNUT2007 nutrient composition database [33]. More details on the development of these codes can be found in the AUSNUT 2007 explanatory notes.

Formulated beverages were defined as all beverages, including caffeinated and non-caffeinated, falling within the “soft drinks and flavoured mineral waters” and “electrolyte, energy and fortified drinks” food code categories [34]. Aggregation of the volume of these beverages consumed (g/day) allowed for the determination of total formulate beverage intake. In addition, the proportion of total soft drink consumed as CFBs was calculated. CFBs were defined as formulated beverages to which caffeine has been added as an ingredient in accordance with the FSANZ Food Standards Code, which restricts caffeine’s use as an additive to cola based beverages and energy drinks [6,7]. Beverages in which caffeine occurs naturally (e.g. tea, coffee, chocolate beverages) were excluded from the CFB definition. The AUSNUT2007 database was examined to determine all beverages consumed on the day of the survey which contained added caffeine, as indicated by the caffeine content of beverages and ingredients list. The amount of these beverages were aggregated to determine total CFB consumption (g/day) as well as caffeine contribution from CFBs (mg/day). Proportion of total caffeine consumed from CFBs was then calculated. Daily caffeine contribution from all beverage sources was determined by aggregating the amount of caffeine consumed from beverages within the “non-alcoholic beverages” food group. This amount was then deducted from total daily caffeine intake, to determine caffeine intake from food sources.

Participants who consumed any CFB (i.e. >0 g) on the day of the survey were classified as a CFB consumer. CFBs were further broken down into different sub-types; including energy drink and non-energy drink (including soft drink and electrolyte beverages) categories. The AUSNUT2007 database was used to define beverages as artificially-sweetened (AS) if labelled ‘intense sweetened’, for example “soft drink, cola flavour, intense sweetened”, which includes beverages marketed as ‘sugar free’ such as Coca-Cola zero or Pepsi max. If this was not specifically defined within the database, for instance “soft drink, cola flavour”, the beverages were assumed to be sugar sweetened (SS), and labelled as such [34].

### Data analysis

All data were analysed using STATA/SE software (version 12.0, Statacorp, College Station, Texas). Statistical significance was set at P < 0.05. To account for the complex sampling frame of the CNPAS the Stata svy command was used, specifying cluster variable (post code), stratum variable (region), and population weightings (age, gender, region).

Frequency (n) and weighted percentages were used to determine the proportion of participants consuming CFBs per capita and stratified by gender, age group, SES, and weight status category. Chi-squared tests were used to assess differences in CFB consumption across categorical variables. Descriptive statistics, mean (95% CI), median (IQR), 90th percentile, and range were used to describe CFB (g/day) (total, AS-CFB and SS-CFB), soft drink (g/day) and caffeine intake (mg/day), in the whole sample as well as stratified by age group, gender and SES. Independent T-tests were used to determine significant differences in total caffeine and energy intakes between consumers and non-consumers of CFB. Histograms and box and whisker plots were used to assess the normality of CFB intake data. On review it was determined that CFB intake (g/day) was highly skewed, hence in addition to reporting CFB consumption per capita, CFB intake was also reported within consumers only.

Within the consumers’ only data, independent t-tests were used to examine differences in intakes by gender and day type, and linear regression analysis was used to assess differences in intakes across age groups and SES categories.

## Results

### Participant characteristics

Table 1 describes the main demographic characteristics and overall energy and caffeine intakes of participants. Overall, 78% of consumers reported consuming caffeine on the day of the survey (data not shown), with 15% of participants classified as CFB consumers. Older children were more likely to consume CFBs than younger children (P < 0.001), and children from a low SES background were more likely to consume CFBs compared with those from a high SES background (P = 0.001, Table 1). There was a significant association between weight status and CFB consumption, whereby CFB consumers were more likely to be overweight or obese (P = 0.008, Table 1). CFB consumers had significantly higher caffeine and energy intakes, compared with non-consumers (P < 0.001, Table 1). Total caffeine intake per kilogram bodyweight was also found to be significantly higher amongst CFB consumers than non-consumers (P < 0.001, Table 1).

**Table 1:** **Demographic characteristics and nutrient intake in Australian children aged 2–16 years by consumption of CFB (n = 4487)**

**Demographic characteristic/Nutrient intake**	**Total sample,** ***n*** **(%)** ^**a**^	**CFB** **consumer,** ***n*** **(%)** ^**a**^	**CFB** **non-consumer,** ***n*** **(%)** ^**a**^	**P** ^**b**^
**No. of participants**	4487	642(15)	3845(85)	
**Gender**				
**Male**	2249(51)	351(55)	1898(50)	0.06
**Female**	2238(49)	291(45)	1947(50)	
**Age group (years)**				
**2-3**	1071(12)	48(3)	1023(14)	<0.001
**4-8**	1216(34)	105(20)	1111(36)	
**9-13**	1110(33)	204(41)	906(32)	
**14-16**	1090(21)	285(36)	805(18)	
**SES category**				
**Low**	1414(30)	253(38)	1161(29)	<0.001
**Mid**	1583(36)	232(37)	1351(36)	
**High**	1490(34)	157(25)	1333(35)	
**Weight classification** ^***c***^				
**Underweight**	212(5)	30(5)	182(5)	0.008
**Healthy weight**	3267(72)	434(66)	2833(74)	
**Overweight**	761(17)	119(19)	642(17)	
**Obese**	247(6)	59(10)	188(5)	
**Energy intake (kJ/day),**				<0.001
**Mean**	8394	9613	8187	
**(95% CI)**	(8250–8537)	(9247–9977)	(8039–8335)	
**Total Caffeine intake (mg/day),**				<0.001
**Mean**	18	61	11	
**(95% CI)**	(17–20)	(55–67)	(10–12)	
**Total Caffeine intake (mg/kg)** ^**d**^				<0.001
**Mean**	0.43	1.33	0.27	
**(95% CI)**	(0.39–0.46)	(1.20-1.45)	(0.25-0.30)	

CFB - Caffeinated, formulated beverage; SES – Socioeconomic Status.

^a^% weighted for age, gender and region; ^b^P values determined using *χ*2 and independent t-test; ^c^Weight classification based on the International Obesity Task Force BMI reference cutoffs [30,31]; ^d^mg/kg bodyweight of participant.

### Dietary sources of caffeine: all participants

**Figure 1:**
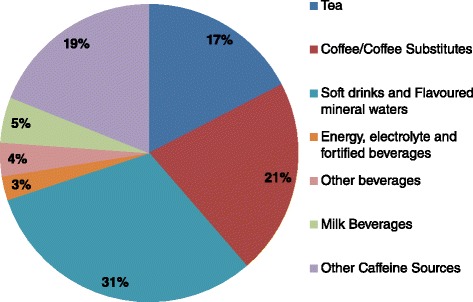
**Dietary sources of caffeine in Australian children aged 2–16 years: total population (n = 4,487).**

Including all participants, mean caffeine intake was 18 mg/day (Table 1). By age group caffeine intakes (mean (95% CI) mg/day) were 3 (2.9-3.8), 8 (7.2-9.0), 19 (17.1-21.4), and 42 (37.3-46.1) in 2–3, 4–8, 9–13, and 14–16 years, respectively. The majority of caffeine was consumed through beverages (81%) and the remainder (19%) from food sources (Figure 1). A brief examination of the AUSNUT2007 database revealed that food sources of caffeine included items such as baked products containing chocolate or cocoa powder. CFBs contributed 34% to daily caffeine intake (Table 2).

**Table 2:** **CFB consumption in Australian children aged 2–16 years, by age group: total population (n = 4487)**

**CFB consumption**	**All (n = 4487)**	**Age group**			
		**2-3 (n = 1071)**	**4-8 (n = 1216)**	**9-13 (n = 1110)**	**14-16 (n = 1090)**
**CFB intake (g/day)**					
**Mean**	64	6	24	79	140
**(95% CI)**	(56–71)	(4–9)	(18–30)	(61–96)	(119–162)
Proportion of caffeine from CFB (%)*	34	17	28	38	33
Proportion of total formulated beverages as CFB (%)*	44	24	34	40	55
Proportion of all CFB as SS-CFB (%)*	77	75	79	80	75
Proportion of all CFB as AS-CFB (%)*	23	25	21	20	25

CFB – Caffeinated, formulated beverage; SS-CFB – Sugar-sweetened caffeinated formulated beverage; AS-CFB - Artificially-sweetened caffeinated, formulated beverage.

*Population proportion data – no measure of variability (i.e. 95% CI) available.

### CFB intake: all participants

Table 2 describes CFB consumption per capita by age group. Average intake was 6 g/day in 2–3 year olds and 140 g/day in 14–16 year olds. The 90th percentiles of intake were 0, 0, 391, and 522 g/day in 2–3, 4–8, 9–13, and 14–16 year age groups, respectively. The proportion of formulated beverages which were caffeinated was higher with increasing age, accounting for just over half of all formulated beverages consumed by 14–16 year olds (Table 2). The majority of CFB consumed was of the sugar-sweetened variety (75-80%).

### CFB intake: CFB consumers only

Table 3 shows beverage consumption data for the CFB consumers (n = 642). Males had significantly higher CFB intakes than females (mean (95% CI) 475 (442–508) g/day vs. 400 (362–437) g/day, P < 0.002). CFB accounted for 83% of all formulated beverages consumed and older children were found to have significantly higher intakes of CFB, compared to younger children (P < 0.001). The majority of CFB consumed was sugar-sweetened, with 75-80% of CFBs consumed as SS-CFB.

**Table 3:** **CFB consumption in Australian children aged 2–16 years, by age group: CFB consumers only (n = 642)**

**CFB consumption**	**All (n = 642)**	**Age group**			
		**2-3 (n = 48)**	**4-8 (n = 105)**	**9-13 (n = 204)**	**14-16 (n = 285)**
**CFB intake (g/day),**					
**Mean**	441	151	287	442	555^a^
**(95% CI)**	(413–469)	(115–187)	(252–321)	(400–484)	(507–602)
**Caffeine from CFB (mg/d)**					
**Mean**	42	14	27	41	55^a^
**(95% CI)**	(39–45)	(11–17)	(22–31)	(37–44)	(48–62)
Proportion of caffeine from CFB (%)*	69	73	74	73	65
Proportion total formulated beverages as CFB (%)*	83	81	86	84	81
Proportion of CFB as SS-CFB (%)*	72	75	79	80	75
Proportion of CFB as AS-CFB (%)*	23	25	21	20	25

CFB – Caffeinated, formulated beverage; SS-CFB – Sugar-sweetened caffeinated, formulated beverage; AS-CFB – Artificially-sweetened caffeinated, formulated beverage.

*Population proportion data – no measure of variability (i.e. 95% CI) available.

^a^Linear regression with age group entered as an indicator variable P < 0.001.

**Figure 2:**
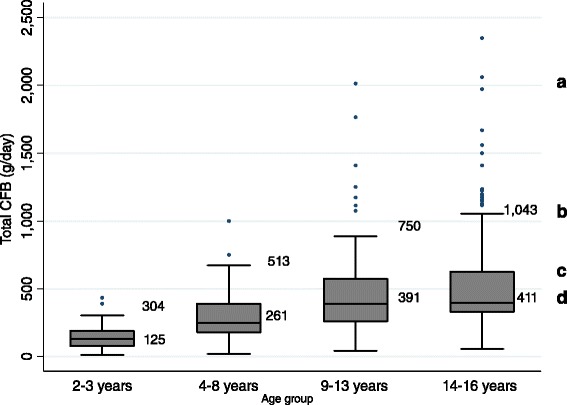
**CFB consumption (g/day) in Australian children aged 2–16 years by age group: CFB consumers only (n = 642).** a: Outlier values; b: 90^th^ Percentile; c: 75^th^ Percentile; d: Median.

Figure 2 shows the range of CFB intakes in consumers only. Median (IQR) intakes were 125 (78–176), 261(170–391), 391 (261–569), and 411 (375–657) g/day for the 2–3, 4–8, 9–13, and 14–16 year old age groups respectively. The 90th percentiles of intake were 304, 513, 750, and 1,043 g/day with maximum intakes reaching 433, 1,000, 2,013, and 2,347 g/day for each of the four age groups respectively. Of the top tenth percentile of CFB consumers (n = 64), median (IQR) intake of total daily caffeine was 104 (82–127) mg/day. The majority (69%) of the consumers within the top tenth percentile fell within the 14–16 year old age group. Within CFB consumers there was no difference in actual intake (g/d) of CFBs consumed across the three SES categories (Table 4, P = 0.2), with the proportion of total formulated beverages as caffeinated beverages also remaining stable (~83%). Approximately 97% of all CFBs were consumed as non-energy drinks (i.e. soft drinks and electrolyte beverages), with 73% consumed as sugar-sweetened non-energy drinks (data not shown).

**Table 4:** **CFB consumption in Australian children aged 2–16 years by socioeconomic status category: CFB consumers only (n = 642)**

**CFB consumption**	**SES category**		
	**Low SES (n = 253)**	**Mid SES (n = 232)**	**High SES (n = 157)**
**CFB intake (g/day)**			
**Mean**	420	461	445
**(95% CI)**	(390–450)	(408–514)	(400–490)
**Caffeine from CFB (mg/day)**			
**Mean**	40	45	42
**(95%CI)**	(37–43)	(39–51)	(38–47)
**% Total Formulated beverages as CFB**	79	86	85

CFB – Caffeinated, formulated beverage; SES – Socioeconomic Status.

### Consumption of CFB by type of day, meal time and location: CFB consumers only

#### Type of day

CFB consumers were significantly more likely to report consuming CFBs on a non-school day compared to a school day (P < 0.001). In addition, the amount of CFB consumed (g/day) was significantly higher on non-school days, when compared to school days (P < 0.005, Table 5).

**Table 5:** **CFB consumption in Australian children aged 2–16 years by type of day: CFB consumers only (n = 642)**

	**School day (n = 292)**	**Non-school day (n = 350)**
**CFB intake (g/day)**		
**Mean**	392	480*
**(95% CI)**	(364–421)	(443–518)

CFB – Caffeinated, formulated beverage.

*T-test: P < 0.005.

#### Meal time

**Figure 3:**
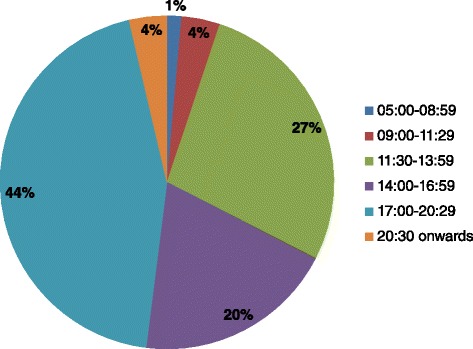
**CFB consumption in Australian children aged 2–16 years by meal time: CFB consumers only (n = 642).**

The majority of CFBs were consumed between 17:00–20:30 (44%), followed by 11:30–13:59 (27%), times traditionally associated with dinner and lunch respectively (Figure 3). When stratified by age group, consumption did not differ from the overall observed trend (data not shown).

#### Location

**Figure 4:**
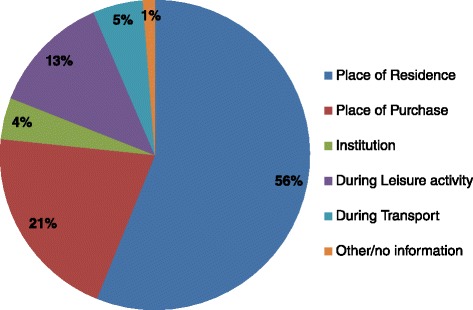
**CFB consumption in Australian children aged 2–16 years by location of consumption: CFB consumers only (n = 642).**

Figure 4 shows CFB consumption by location. Place of residence was the most popular location of consumption with ~56% of all CFBs consumed here, followed by consumption at a leisure activity (21%) (Figure 4). When stratified by age group, older children had a greater variety in their location of consumption with increased consumption during transport and leisure activities, however place of residence remained the predominant location of consumption across all age groups (data not shown).

## Discussion

These are the first data available describing consumption patterns of CFBs in a nationally representative sample of Australian children and adolescents. It is evident that consumption of CFBs occurs across all ages (2–16 years) and significantly increased total caffeine intake in children and adolescents. Although the data revealed that older children were more likely to consume CFBs, consumption still occurred in the younger age groups with 4% of 2–3 year olds and 8% of 4–8 year olds consuming CFBs on the day of the survey. The majority of CFB consumption occurred within the home environment between 17:00–20:30 on non-school days. In the present study children of a low socioeconomic background were significantly more likely to consume CFBs, a result which echoes previous findings of research conducted on SSB consumption using the same sample of Australian children and adolescents [35,36].

It has previously been hypothesized that the addition of caffeine in beverage formulation may lead to increased consumption of beverages associated with adverse health outcomes [26,27]. A recent randomised control trial by Keast et al. supports this hypothesis whereby the inclusion of caffeine within a SSB resulted in a significant increase in SSB consumption despite study participants’ inability to detect any taste difference between beverages [26]. Within the present study the most commonly consumed form of CFBs were sugar-sweetened, non-energy drinks. Consumers of CFB were found to have higher energy intakes and were more likely to be overweight or obese. SSBs have been associated with overweight and obesity [20-23]. Previous work has indicated that the addition of caffeine to formulated beverages results in increased consumption of SSB and removal of caffeine may result in reduced energy intake from SSB in this age group [26,27]. As 44% of all formulated beverages consumed within the current study were caffeinated, removing or decreasing caffeine in beverage formulation has the potential to decrease consumption of these beverages. Over time this may help to counter the trajectory of unnecessary weight gain in children and adolescents [27].

It was also evident that CFB consumers had significantly higher caffeine intakes than non-consumers, indicating that CFB consumption significantly increases total caffeine intake. Therefore, reducing the permissible level of caffeine as an additive in beverage formulation could reduce overall caffeine intake in children and adolescents. Although the total caffeine intakes observed within the current study are below recommended intake levels per kilogram of bodyweight [2], these data represent intakes from 2007. Changes in formulated beverage production and marketing have occurred since this time, with a wider variety of CFBs available within the Australian market and an increase in portion sizes also evident [8]. Therefore, it is important that the consumption of CFBs continue to be monitored to ensure that the practice of adding caffeine to commonly consumed beverages available to children and adolescents does not put them at risk of exceeding these recommendations. The results from this study provide important baseline data for tracking changes in consumption over time. By monitoring consumption patterns over time, potential areas for intervention can also be identified.

### Potential areas for intervention

Identifying the demographic factors associated with consumption of CFBs may help to identify intervention points. Our results reflect those of a similar study conducted within the same population group by Hafekost et al. (2011), who found that older children were more likely to consume SSBs and that consumption of these beverages was highest at home [36]. Similarly, in the present study we observed that 56% of CFBs were consumed at a place of residence and 44% of CFBs were consumed within the “dinner” time bracket, where parental involvement in food choices are likely. In addition to modifying current food regulations that permit the inclusion of caffeine as an additive in beverage formulation, the results from this study suggest the need to target parental influences over beverage choices with meals. As research has shown that parental food choices can significantly influence food and beverage consumption in children through both exposure and role-modelling [37,38], targeting parental acceptance of CFBs as a mealtime beverage and promoting alternative beverage options such as water and unflavoured milk may lower children’s consumption of CFBs. These interventions should be in a form that is accessible and meaningful to older children and adolescents across a range of SES backgrounds.

### Limitations of this research

The main strength of this study is the use of data from the Australian National Nutrition and Physical Activity Survey, a large nationally representative sample of Australian children and adolescents. However, despite the robust sampling procedures used, participation was voluntary and thus some differences exist between the sample population and the broader Australian population; participants were typically from higher income earning households, than the average population [28]. The use of 24-hour recalls also provides some limitations as they are subject to participant recall bias and underreporting [39]. However previous analyses of the 2007 CNPAS data indicated that under-reporting in this sample of children was only evident in 14–16 year olds, and the extent of under-reporting within this age group was proven to be minimal [29].

Data for this study was collected over a six-month period from February to August, encompassing three different seasons. In Australia this covers summer, autumn and winter. Although this minimises the possible effect of seasonality on results, it should be noted that seasonal variation may influence fluid consumption and this may limit the results of this study. Due to the wide age range of participants within the survey and the lack of participant defined meal times, some misclassification of meal times may have occurred and consumption patterns reported across meal time may have been incorrectly estimated.

## Conclusions

Consumption of CFBs occurred across all age groups and was associated with increased energy and caffeine intakes. Modifications to the permissibility of caffeine as a food additive may be an appropriate strategy to reduce the intake of caffeine and CFB in this age group. These data provide insights into potential intervention targets to lower intakes of CFBs, including parental influences over beverages choices with meals. These interventions should be in a form that is accessible and meaningful to older children and adolescents across a range of SES backgrounds.

## Abbreviations

CFB: Caffeinated, formulated beverage
SSB: Sugar-sweetened beverage
SS-CFB: Sugar-sweetened caffeinated, formulated beverage
ASB: Artificially-sweetened beverage
AS-CFB: Artificially-sweetened caffeinated, formulated beverage
FSANZ: Food standards Australia New Zealand
CNPAS: Children’s nutritional and physical activity survey
SES: Socioeconomic status
AHS: Australian Health Survey
